# Genome-wide association study identifies *CBFA2T3* affecting the rate of CSF Aβ_42_ decline in non-demented elders

**DOI:** 10.18632/aging.102125

**Published:** 2019-08-01

**Authors:** Kai-Xin Dou, Can Zhang, Chen-Chen Tan, Wei Xu, Jie-Qiong Li, Xi-Peng Cao, Lan Tan, Jin-Tai Yu, for the Alzheimer’s Disease Neuroimaging Initiative (ADNI)

**Affiliations:** 1 Department of Neurology, Qingdao Municipal Hospital Affiliated to Qingdao University, Qingdao, China; 2 Genetics and Aging Research Unit, MassGeneral Institute for Neurodegenerative Diseases (MIND), Department of Neurology, Massachusetts General Hospital and Harvard Medical School, Charlestown, MA 02129, USA; 3 Clinical Research Center, Qingdao Municipal Hospital, Qingdao University, Qingdao, China; 4 Department of Neurology and Institute of Neurology, Huashan Hospital, Shanghai Medical College, Fudan University, Shanghai, China

**Keywords:** Alzheimer’s disease, amyloid, cerebrospinal fluid, genetics, GWAS

## Abstract

Brain amyloid deposition is an early pathological event in Alzheimer’s disease (AD), and abnormally low levels amyloid-β_42_ peptide (Aβ_42_) in cerebrospinal fluid (CSF) can be detected in preclinical AD. To identify the genetic determinants that regulate the rate of CSF Aβ_42_ decline among non-demented elders, we conducted a genome-wide association study involved 321 non-demented elders from Alzheimer’s Disease Neuroimaging Initiative (ADNI) 1/GO/2 cohorts restricted to non-Hispanic Caucasians. A novel genome-wide significant association of higher annualized percent decline of CSF Aβ_42_ in the gene *CBFA2T3* (*CBFA2/RUNX1* translocation partner 3; rs13333659-T; *p* = 2.24 × 10^−9^) was identified. Besides displaying abnormal CSF Aβ_42_ levels, rs13333659-T carriers were more likely to exhibit a greater longitudinal cognitive decline (*p* = 0.029, *β* = 0.097) and hippocampal atrophy (*p* = 0.029, *β* = −0.160) in the non-demented elders, especially for the participants who were amyloid-positive at baseline. These findings suggest rs13333659 in *CBFA2T3* as a risk locus to modulate the decline rate of CSF Aβ_42_ preceding the onset of clinical symptoms.

## INTRODUCTION

Amyloid-β pathology is the starting event in the serial biomarker cascade models of Alzheimer’s disease (AD), which precedes abnormality in clinical symptoms for almost two decades and subsequently contributes to clinical progression [1, 2]. Approximately one-fourth of cognitively normal populations and half of individuals with mild cognitive impairment (MCI) have elevated levels of cerebral amyloidosis [3, 4]. The 2018 National Institute on Aging and Alzheimer’s Association (NIA-AA) research framework updated the biomarker classification system consisting of β*-*amyloidosis, tauopathy and neurodegeneration or neuronal injury (the ATN system) and emphasized the presence of amyloid pathology to identify the first stage of the disease [5, 6]. Abnormality of amyloid-β precedes abnormality in tau, which leads to AD–related brain atrophy, [^18^F]-fluorodeoxyglucose (FDG) decline and accelerated cognitive decline [7].

AD is a defined biological entity with a progressive continuum in clinical abnormalities. The molecular pathology of AD is accomplished by biological entity-related continuous measures, and highlighted by three disease components, including β*-*amyloidosis, tauopathy and neurodegeneration. Importantly, quantifications of amyloid-β have been incorporated into standard diagnostic guidelines and clinical trials, and abnormal amyloid-β can be measured by decreased cerebrospinal fluid (CSF) amyloid-β_42_ peptide (Aβ_42_) levels or increased ligand retention of amyloid positron emission tomography (PET) imaging in the preclinical phase [8–11]. Previous cross-sectional and longitudinal studies have indicated that abnormally low levels of CSF Aβ_42_ can be detected before the identification of abnormal Aβ_42_ by PET in preclinical AD [12, 13]. Consequently, the reduction in CSF Aβ_42_ levels over time has stronger predictive value for identifying population with the disease or condition and monitoring disease trajectories at early stage.

Genetic risk factors play a key role in disease pathology with a high heritability of 70% to 80% [14]. The apolipoprotein E *(APOE)* genotype is regarded as the strongest genetic regulator for amyloid pathology in late-onset AD. However, at present, the genetic risk factors that are related to longitudinal changes of CSF Aβ_42_ levels remain poorly understood. Use of quantitative traits in genome-wide association studies (GWAS) provides novel and important insights into broader trends in associations with genes and related pathways [15–17]. Therefore, we performed a quantitative trait GWAS with longitudinal measures of CSF Aβ_42_ levels from the Alzheimer’s Disease Neuroimaging Initiative (ADNI) database in order to identify genetic regulators of CSF Aβ_42_ change rate in non-demented elders**.**

## RESULTS

### Characteristics of included subjects

The final participants were consolidated for 321 non-Hispanic Caucasian subjects from the ADNI-1, 2 and Grand Opportunities (GO) databases. Participants included a mixture of 195 MCI and 126 cognitively normal subjects. The mean duration from the first measurement of CSF Aβ_42_ to the last was 31.3 months. Table 1 shows the distribution of baseline demographic information. No significant differences in gender (*p* = 0.11) and education level (*p* = 0.11) were found between the two diagnostic groups, while age, cognitive score, *APOE* genotype and CSF Aβ_42_ level differed between groups (*p* < 0.001).

**Table 1:** Demographic information and clinical characteristics of participants at baseline.

**Baseline diagnosis**	**CN**	**MCI**	***P* value**
Participants (n)	126	195	
Baseline age, y, mean (SD)	75.06 (5.41)	72.05 (7.19)	<0.001
Gender (male/female)	66/60	120/75	0.11
Education, y, mean (SD)	16.68 (2.75)	16.19 (2.78)	0.11
*APOE* (ε4−/ε4+)	102/24	101/94	<0.001
CSF Aβ_42_, mean (SD)	203.52 (57.00)	171.86 (53.09)	<0.001
MMSE scores, mean (SD)	29.17 (1.13)	27.79 (1.75)	<0.001
ADAS-cog11 scores, mean (SD)	5.69 (2.72)	9.83 (4.22)	<0.001
Hippocampus, mm³, mean (SD)	7081.30 (1080.34)	7078.58 (1083.55)	<0.001

Abbreviations: Aβ_42_ = amyloid-β_42_ peptide; CSF = cerebrospinal fluid; CN = cognitively normal; MCI = mild cognitive impairment; *APOE ε*4 = apolipoprotein E *ε*4 allele; MMSE = Mini-Mental State Examination; ADAS-cog = Alzheimer’s disease assessment scale-cognitive subscale.

*P* values are from the Wilcoxon rank-sum test

### GWAS of longitudinal changes in CSF Aβ_42_

**Figure 1:**
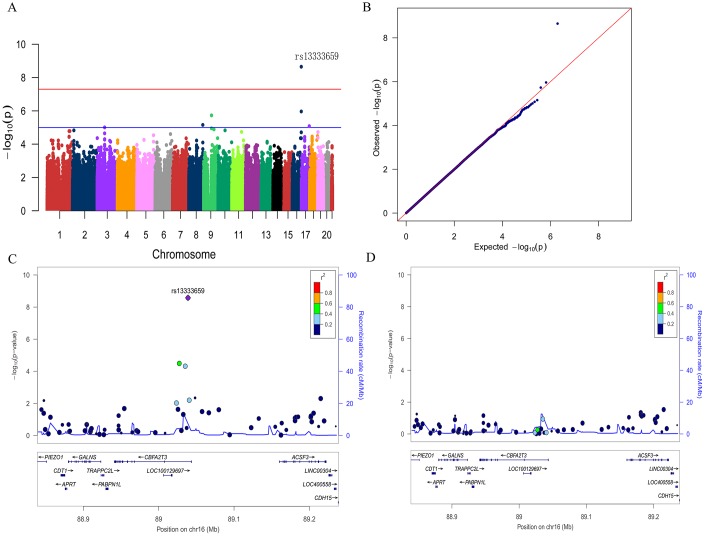
Manhattan plot (**A**), quantile-quantile plot (**B**) and regional plots (**C** and **D**) for the GWAS of longitudinal changes of CSF Aβ_42._ (**A**) A genome-wide significant association (P < 5×10^−8^; red line) with longitudinal change of CSF Aβ_42_ was identified on chromosome 16 within *CBFA2T3*. Suggestive associations are at the threshold of P < 1×10^−5^ (blue line). (**B**) Quantile-quantile plot. (**C**) Regional association results for the 88.9 Mb to 89.2 Mb region of chromosome 16. (**D**) Association results for the 88.9 Mb to 89.2 Mb region of chromosome 16 controlling for rs13333659.

**Figure 2:**
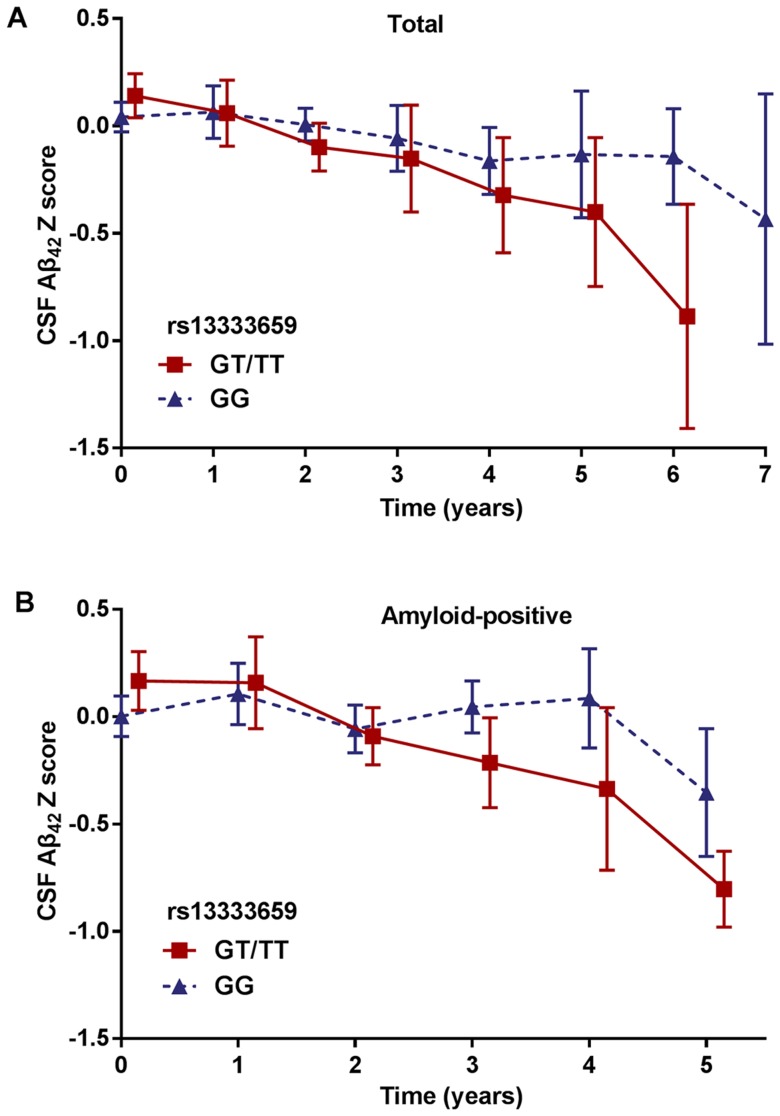
**Mean changes of CSF Aβ_42_ over time in rs13333659 minor allele carriers vs. non-carriers.** (**A**) Mean concentrations of CSF Aβ_42_ ± SE (standard error) change for rs13333659 minor allele carriers vs. non-carriers in total GWAS cohort during follow-up period. (**B**) Mean concentrations of CSF Aβ_42_ ± SE change for rs13333659 minor allele carriers vs. non-carriers in baseline amyloid-positive group during follow-up period.

To discover novel genetic modifiers of annualized percent change in CSF Aβ_42_, we conducted a GWAS adjusting for age, gender, education level, disease status (MCI or CN), *APOE ε*4 status, follow-up duration and two principal component factors. The strongest association of the longitudinal changes in CSF Aβ_42_ was identified with rs13333659, an intronic variant located in the gene *CBFA2T3* (*CBFA2/RUNX1* translocation partner 3; OMIM*603870, 16q 24.3; Figure 1A). Figure 2A shows the mean concentration of CSF Aβ_42_ during the follow-up period for the minor allele (T) carriers vs. non-carriers in the discovery cohort and baseline amyloid-positive group. There are 214 individuals who were homozygous wild-type (G/G), 98 individuals who were heterozygous (G/T) and 9 individuals who were homozygous recessive (T/T) at rs13333659. The minor allele T (MAF = 0.1) was associated with a more rapid decline in CSF Aβ_42_ levels over time (*β* = −0.034, *p* = 2.24×10^−9^). This novel statistically significant association was shown in both baseline amyloid-positive (*β* = −0.034, *p* = 1.95×10^−6^ Figure 2B) and amyloid-negative (*β* = −0.027, *p* = 2.74×10^−3^) groups.

We then utilized the Q-Q plot to compare the observed distribution and the expected distribution for the GWAS (Figure 1B). There was a deviation from expected *p*-values in the upper tail distribution, suggesting the presence of a significant genetic association. The genomic inflation factor λ was 1.007, indicating no evidence of significant confounding due to population stratification. The Figure 1C shows the linkage disequilibrium (LD) between rs13333659 in *CBFA2T3* and other nearby SNPs, several of which showed strong associations with the longitudinal changes of CSF Aβ_42_ (*p* < 0.01). No significant associations were observed after controlling for rs13333659 genotype, indicating that these associations for nearby SNPs were driven by rs13333659 (Figure 1D). Five suggestive associations (*p* < 1 × 10^−5^) with longitudinal changes of CSF Aβ_42_ were demonstrated in this GWAS (Figure 1A and Table 2), which included an additional SNP in *CBFA2T3* (rs57706252), *TRPM6* (rs35878400, OMIM*607009), *RBFOX3* (rs113685315, OMIM*616999), PDZRN3 (rs55732227, OMIM*609729) and an intergenic locus on 8q21.1 (rs6987191).

**Table 2:** Peak SNPs associated with annualized percent change of CSF Aβ_42_.

**CHR**	**SNP**	**Gene**	**Position**	**Function**	**A1/2**	**MAF**	***β*^a^**	***P* value**
16	rs13333659	*CBFA2T3*	89038880	Intron	T/G	0.177	−0.034	2.24×10^−9^
16	rs57706252	*CBFA2T3*	89030609	Intron	C/T	0.167	−0.029	1.09×10^−6^
9	rs35878400	*TRPM6*	77482795	Intron	T/C	0.273	0.024	1.87×10^−6^
8	rs6987191	/	138246221	/	C/T	0.131	−0.031	6.99×10^−6^
17	rs113685315	*RBFOX3*	77390248	Intron	A/G	0.162	0.025	8.31×10^−6^
3	rs55732227	*PDZRN3*	73611629	Intron	T/A	0.309	−0.020	9.86×10^−6^

Abbreviations: CHR = chromosome; SNP = single nucleotide polymorphism; MAF = minor allele frequency; A1/2 = minor allele/major allele.

^a^ *β* effect size from GWAS means the annualized percent change of CSF Aβ_42_ conferred by one copy of the minor allele.

### Impact of *CBFA2T3* on longitudinal cognitive performance

**Figure 3:**
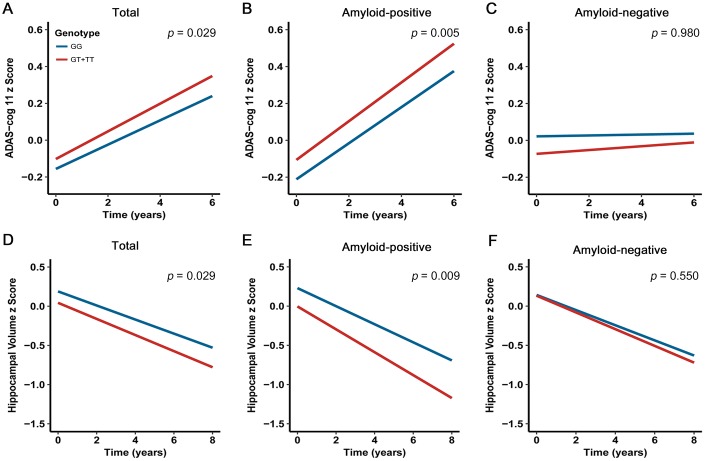
**Effects of CBFA2T3 rs13333659-T on cognitive performance and hippocampal volume over time.** Data from linear mixed-effects models adjusted for age, gender, educational level, *APOE* ε4 genotype, disease status, follow-up duration, as well as intracranial volume for hippocampal volume. ADAS-cog 11 indicates Alzheimer Disease Assessment Scale-cognitive subscale.

Amyloid aggregation confers the greatest risk for steeper long-term cognitive decline [18]. Several recent studies have confirmed that the negative effects of amyloidosis on cognitive performance in cognitively normal individuals, which are distinct from the influence of aging [18–21]. Taken together, we hypothesized that carriers with rs13333659-T would display a higher rate of cognitive decline. In order to assess the relationship between rs13333659 variants and cognitive decline over time, we performed a linear mixed effects analysis on 687 non-demented elderly subjects (MCI = 433; CN = 254) from the ADNI cohort with a 6-year follow-up. The cognitive assessments were based on ADAS-cog 11 scale. After controlling for genotype, age, gender, education level, disease status, *APOE* genotype and follow-up duration, we found rs13333659-T carriers were associated with accelerated decline in cognitive performance compared to non-carriers in all non-demented samples (*p* = 0.029, *β* = 0.097, n = 687; Figure 3A). The effects were more significant in baseline amyloid-positive group (*p* = 0.005, *β* = 0.196, n = 297; Figure 3B) but not in baseline amyloid-negative group (*p* = 0.980, *β*= 0.003, n = 240; Figure 3C). These data supported the association between the minor allele (T) of rs13333659 and a more rapid cognitive decline, related to amyloid status.

### Impact of *CBFA2T3* on longitudinal brain structure

The emerging amyloid pathology has been demonstrated to result in atrophy rates of brain structures during the development of AD [22–24]. We hypothesized that rs13333659-T carriers in *CBFA2T3* would show faster rates of atrophy in AD-related brain regions (such as the hippocampus region). In order to assess the relationship between rs13333659 variants and hippocampal atrophy over time, we examined longitudinal changes of hippocampal volume over 8 years using linear mixed models. Five hundred and eighty-six non-demented elders (MCI = 361, CN = 225) with structural MRI data from ADNI were examined in this longitudinal analysis. Our analyses were conducted when genotype, age, gender, education level, disease status, *APOE ε*4 genotype, follow-up duration and intracranial volume were included as covariates. The results revealed that rs13333659-T carriers have increased hippocampal atrophy rates compared to non-carriers across all subjects (*p* = 0.029, *β*= −0.160, n = 586; Figure 3D). In particular, the higher rate of hippocampal atrophy was observed in baseline amyloid-positive group (*p* = 0.009, *β* = −0.285, n = 260; Figure 3E), while no significant differences were observed between rs13333659-T carriers and non-carriers in baseline amyloid-negative group (*p* = 0.550, *β* = −0.083, n = 207; Figure 3F).

### DISCUSSION

In a series of longitudinal measurements of CSF Aβ_42_, we have identified a genome-wide significant association of *CBFA2T3* rs13333659-T with a more rapid decline rate of CSF Aβ_42_ in non-demented elders. The novel association for the SNP may reflect a relative specificity for amyloid versus the more heterogeneous case-control status, increasing power obtained via endophenotype analysis. This novel association was further validated by converging evidence from cognitive performance and brain MRI structure analyses with consistent downtrends in a longitudinal framework. Our findings provide evidence that *CBFA2T3* as a novel candidate gene may promote the application of CSF Aβ_42_ as an early biomarker to the prediction of the conversion from cognitive health to AD and the detection of disease trajectories.

*CBFA2T3* encodes a myeloid translocation gene (*MTG*) family protein with highly conserved sequences across species, which interacts with transcription factors of DNA-bounding sites and recruits multiple co-repressors to facilitate transcription repression [25]. The gene expresses during the process of neurogenesis and neuronal differentiation, which may function to promote the transition from precursor to neuron and the expression of neuronal genes in differentiated cells [26]. The putative dominant-negative mutant of the gene causes a large number of differentiated neurons markedly reduced [26]. Prior to this study, the definite association mechanism of CSF Aβ_42_ and *CBFA2T3* has not been previously been discovered. The expression of *CBFA2T3, NEUROG2* (OMIM*606624) and *ASCL1* (OMIM*100790) genes overlapped, and transcription assay showed that *NEUROG2* and *ASCL1* are inhibited by the *MTG*protein family, in which the *CBFA2T3* gene was the most efficient to inhibit the transcription activity [27]. The proneural factor *NEUROG2* mediates *APP*-stimulated neuronal differentiation of neural stem/progenitor cells (*NSPCs*) to neurons, and levels of *NEUROG2* are correlated with amyloid-precursor protein (*APP*) expression [28], which means that rare mutations of *CBFA2T3* might influence the *APP* expression. *APP* is a single-pass transmembrane protein expressed at high levels in the brain, and the generation and accumulation of Aβ_42_ from sequential *APP* proteolysis is the crucial step in the development of AD [29]. However, the specific mechanism through which *NEUROG2* regulates *APP* is not yet clear. Furthermore, *NEUROG2* expression increased with rising relative Aβ_42_ levels [30]. The recent findings from Alzheimer’s disease mouse models revealed that lentiviral vector-mediated overexpression of *NEUROG2* and *ASCL1* can ameliorate learning and memory impairment, and the *ASCL1*gene treatment could be linked to inhibition of the neuroinflammatory response and enhancement of neuroprotection and neurogenesis [31]. Taken together, we reasoned that *CBFA2T3* might affect the neurogenesis process and AD-related cognitive decline.

The Aβ is a product of the metabolism of the amyloid precursor protein, a type 1 transmembrane protein [32]. *APP* can mediate cell adhesion and stimulate neurite outgrowth. Moreover, a secreted form of *APP* stimulates cell proliferation and is neuroprotective against a variety of toxic insults, including oxidative stress, glucose deprivation and excitotoxicity [33]. An imbalance between the production and clearance of Aβ has a crucial role in the development and progression of AD. The toxic soluble Aβ oligomers (AβOs) accumulate in AD brain and constitute long-lived alternatives to the disease-defining Aβ fibrils deposited in amyloid plaques [34]. The β*-*amyloidosis triggers a redistribution of critical synaptic proteins and induces hyperactivity in metabotropic and ionotropic glutamate receptors. This leads to Ca^2+^ overload and instigates major facets of AD neuropathology, including tau hyperphosphorylation, insulin resistance, oxidative stress, synapse loss and so on [35]. The AβO hypothesis has been described as a small conceptual revolution and is widely regarded as accounting for the onset of neuron damage leading to AD.

Suggestive associations were identified through GWAS and might reach genome-wide significance in larger cohorts of non-demented older adults. The suggestive associations included a SNP in *RBFOX3*, which is exclusively expressed in neurons. *RBFOX3* promotes neuronal differentiation through alternative splicing of *Numb* pre-mRNA during brain development and maturation [36]. *RBFOX3* mutations have been observed to be linked to cognitive impairments. Moreover, *RBFOX3* is critical for normal hippocampal function. Especially in hippocampal *CA1* and dentate gyrus regions, the dysfunctional *RBFOX3* affects synaptic transmission and short-time or long-time plasticity and results in impairment of learning, memory and cognition [37, 38]. The suggestive findings argue for further investigation into *RBFOX3* to clarify the potential functional variants related to amyloid pathology.

Following this GWAS scan, we assessed the associations of *CBFA2T3* rs13333659-T with other AD-related endophenotypes in the longitudinal framework. In particular, it was observed that rs13333659-T carriers had more rapid rates of cognitive decline and hippocampal atrophy among the non-demented elders with detectable amyloid pathology at baseline. It has been validated by previous studies that amyloid status can be present in the brain and has a minor impact on cognition over the long preclinical period [20, 39, 40]. Our results also suggest that baseline amyloidosis as an enrolling criterion in many prevention trials might be useful for risk enrichment and risk stratification [41]. The CSF Aβ_42_ (or the Aβ_42_/Aβ_40_ ratio) and amyloid-PET were widely accepted as measurable indicators of abnormal pathological states associated with cerebral amyloidosis levels [42]. The updated 2018 NIA-AA research framework indicates that focusing on longitudinal cohort studies and randomized placebo-controlled trials facilitate the biological definition of AD [5]. In the absence of successful anti-amyloid trials, the clearly identifiable stages and mechanisms behind the efficacy of the anti-amyloid treatment remain unknown. Probably the most effective approach would be to find preventive targets to inhibit the initial accumulation of Aβ_42_. Thus, understanding the genetic mechanisms underlying the rate of amyloid aggregation in individuals without dementia may be the best window on a better understanding of the pathological changes in AD and the design of prevention trials [43]. This study discovered a novel risk locus which can influence the decline rate of CSF Aβ_42_ decline preceding the onset of clinical symptoms, providing reliable and valid estimates for further investigations.

Our findings must be interpreted in the light of the limitations. Although we leveraged publicly available ANDI genetics and longitudinal CSF Aβ_42_ data to perform this original study, the sample size for analysis was relatively small, leading to the limited power to identify variants which have small effects. Given that the buildup of brain Aβ_42_ is postulated to take decades, the follow-up of this study is not sufficiently long to monitor longitudinal changes. Replication testing is necessary in larger samples with long duration of follow-up to confirm our findings and additional discovery. The study was limited to non-Hispanic Caucasian participants to avoid population stratification across ethnicities, but the rs13333659 in *CBFA2T3* has various frequencies in different races. The contradiction determines the racial limitation of our research and the necessity of replication analysis in other races. Furthermore, this study provided evidence for association between rs13333659 in *CBFA2T3* genetic variants with the decline rate of CSF Aβ_42_ as well as that of rs13333659 and cognitive performance traits, but the specific mechanisms of influencing expression level or protein structure and affecting phenotypes were not illustrated. Functional genomics experiments have not yet been performed, which included immunohistochemistry and analyses of *CBFA2T3* knockout models.

## METHODS

### Participants

In this study, the final participants (n = 321) included cognitively normal subjects (n = 126) and patients with MCI (n = 195) after all quality control. All participants were enrolled from the ADNI 1, ADNI 2 and ADNI GO phases. The ADNI was launched in 2003 as a public-private partnership, led by Principal Investigator Michael W. Weiner, MD. The primary goal of ADNI has been to test whether serial magnetic resonance imaging (MRI), PET, other biological markers, and clinical and neuropsychological assessment can be combined to measure the progression of MCI and early AD. For up-to-date information see http://www.adni-info.org.

**Figure 4:**
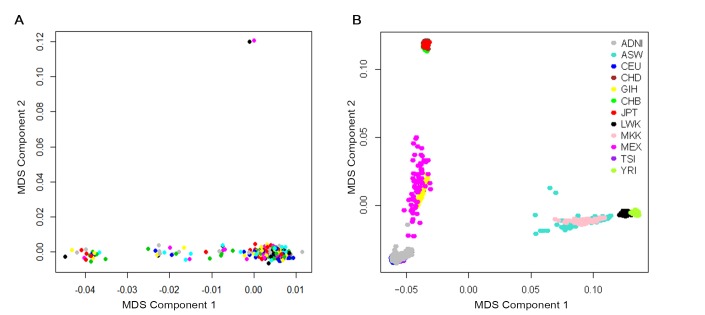
**Cryptic relatedness and population stratification checked with genomic identity-by-descent (IBD) and multidimensional scaling (MDS) components.** (**A**) MDS plot of ADNI non-Hispanic White samples. Two samples were outliers based on the second MDS component (at above of plot; 024_S_2239 and 024_S_4084). (**B**) MDS plot of ADNI samples overlaid on HapMap samples. The ancestry of the HapMap participants is shown by the point color. Abbreviations: ADNI: Alzheimer’s Disease Neuroimaging Initiative; ASW: African ancestry in Southwest USA; CEU: Utah residents with Northern and Western European ancestry from the CEPH collection; CHB: Han Chinese individuals from Beijing, China; CHD: Chinese in Metropolitan Denver, Colorado; GIH: Gujarati Indians in Houston, Texas; JPT: Tokyo, Japan; LWK: Luhya in Webuye, Kenya; MEX: Mexican ancestry in Los Angeles, California; MKK: Maasai in Kinyawa, Kenya; TSI: Tuscans in Italy; YRI: Yoruba in Ibadan, Nigeria.

A total of 370 participants with longitudinal CSF Aβ_42_ data and genotype data were available before quality control. Participants were restricted to non-demented elders and we removed 13 patients diagnosed with AD. To address confounding due to population stratification, we restricted the participants to non-Hispanic Caucasians and this step removed 33 subjects. To reduce potential bias due to due to family structure and cryptic relatedness, we checked the preliminary results with genomic identity-by-descent (IBD) and multidimensional scaling (MDS) components using PLINK v1.9 software. Two samples were outliers based on the second MDS component and the identity-by-descent fraction (π) of these two was equal to 0.43, suggesting they are probably first-degree relatives (Figure 4A). This step removed these two individuals. Finally, using the HapMap cohort, they showed tight clustering with individuals of European ancestry (Figure 4B).

### CSF measurements

Cerebrospinal fluid was acquired by lumbar puncture and stored at −80°C in the ADNI Biomarker Core laboratory at the University of Pennsylvania Medical Center. CSF Aβ_42_ was measured using the xMAP Luminex platform and Innogenetics/Fujirebio AlzBio3 immunoassay kits. Additional information can be found at http://adni.loni.usc.edu/methods/biomarker-analysis. Longitudinal CSF Aβ_42_ information was collected in this GWAS with at least one year follow-up measurements. The annualized percent changes in CSF Aβ_42_ were based on the measurements in each follow-up. The annualized percent changes in CSF Aβ_42_ were calculated as follows:

$$Y=\frac{1}{n-1}\sum_{k=2}^{n}\left(\frac{{\text{a}}_{\text{k}}-{\text{a}}_{\text{k}-1}}{{\text{a}}_{\text{k}-1}}\right)$$

Where Y= the annualized percent changes in CSF Aβ_42_; n = the number of measurements in CSF Aβ_42_ (when n = 1 indicates the baseline measurement); a_k_= the CSF Aβ_42_ level at the k^th^ measurement (a_1_ indicates the baseline level). Extreme outliers (annualized percentage change > four standard deviations from the sample mean) were excluded to limit the potential bias for spurious associations. This step removed one additional individual, resulting 321 available subjects. Participants were categorized into amyloid-positive and amyloid-negative groups using the cut-off values that were ± 5% from the original cut-offs to avoid borderline effects [44, 45]. The cut-offs used in this study were: CSF amyloid-positive < 182.4ng/L, CSF amyloid-negative > 201.6ng/L; PET amyloid-positive > 1.1655 standardized uptake value ratio (SUVR), PET amyloid-negative < 1.0545 SUVR.

### Genotyping and quality control

Genotyping was performed according to the manufacturer’s protocol using blood DNA samples with the Illumina Human610-Quad BeadChip (Illumina Inc) or theIllumina HumanOmniExpressBeadChip (Illumina Inc). Stringent quality control assessment was performed on samples using the PLINK v1.9 software with the following criteria: minimum call rate for SNPs and subjects > 95%, minimum allele frequencies (MAF) > 0.1, Hardy-Weinberg equilibrium (HWE) test *p* > 0.001. The final data set included 1040042 SNPs and 321 participants. APOE alleles which were defined by rs7412 and rs429358 were genotyped separately by an *APOE* genotyping kit [17].

### Statistical analysis

GWAS was performed using liner regression analysis under the assumption of the additive genetic model in PLINK v1.9 software. Covariates included baseline age, genders, education level, baseline diagnosis (MCI or CN), follow-up duration and *APOE ε*4 status and two principal component factors. We included APOE *ε*4 as a covariate in the GWAS for limiting the effects on *APOE ε*4 genotype. The suggestive association threshold was *p* < 1×10^−5^and the conservative significance threshold for genome-wide significance was *p* < 1×10^−8^ [46]. The Manhattan plot and Q-Q plot were generated with the qqman package in R software (Version 3.4.3). Regional association plots were obtained using LocusZoom web tool (http://locuszoom.org/).

Additional analyses were performed to test the association between genotypes of the top SNP and longitudinal changes in other AD-related phenotypes (included cognitive performance and hippocampal structure) for ADNI participants using linear mixed effects model implemented in R software (version 3.4.3). The linear mixed model interprets the correlation structure with in each time sequence of measurements in each participant, and it permits different follow-up periods and varying rates among individuals. Longitudinal changes in cognitive performance were assessed by Alzheimer Disease Assessment Scale-cognitive subscale (ADAS-cog 11). This assessment scale is known to be sensitive measures of cognitive decline and it was acquired longitudinally from the ADNI dataset. Longitudinal changes of hippocampus volume were acquired using T1-weighted structural MRI scans with a sagittal volumetric magnetization-prepared rapid acquisition gradient echo sequence. All outcome variables in linear mixed model were standardized to z scores to facilitate comparisons between modalities, and statistical significance was set at the threshold of *p* < 0.05.

## CONCLUSIONS

In conclusion, a genome-wide significant SNP, rs13333659 in *CBFA2T3* gene is associated with a more rapid decline rate of CSF Aβ_42_ in non-demented elders. This study provides additional evidence that the minor allele (T) of rs13333659 displays negative effects on other AD-related endophenotypes in the longitudinal framework, including more poor cognitive performance and smaller hippocampal volume. The gene has not been identified to have association with amyloid deposition in the brain, but it is involved in neurogenesis and neuronal differentiation. Further investigation is warranted to explore theprecise functional pathways underlying the mechanisms conferred by *CBFA2T3* for disease progression. More importantly, further validation of this novel genetic association in large samples and different populations may further support *CBFA2T3*-related pathways as a potential target for improving both risk stratification and preventive and therapeutic development.
